# High-performance metal mesh/graphene hybrid films using prime-location and metal-doped graphene

**DOI:** 10.1038/s41598-017-10355-5

**Published:** 2017-08-31

**Authors:** Jung-Hong Min, Woo-Lim Jeong, Hoe-Min Kwak, Dong-Seon Lee

**Affiliations:** 1 School of Electrical Engineering and Computer Science, Gwangju Institute of Science and Technology, Gwangju, 61005 South Korea; 2 Research Institute for Solar and Sustainable Energies, Gwangju, 61005 South Korea

## Abstract

We introduce high-performance metal mesh/graphene hybrid transparent conductive layers (TCLs) using prime-location and metal-doped graphene in near-ultraviolet light-emitting diodes (NUV LEDs). Despite the transparency and sheet resistance values being similar for hybrid TCLs, there were huge differences in the NUV LEDs’ electrical and optical properties depending on the location of the graphene layer. We achieved better physical stability and current spreading when the graphene layer was located beneath the metal mesh, in direct contact with the p-GaN layer. We further improved the contact properties by adding a very thin Au mesh between the thick Ag mesh and the graphene layer to produce a dual-layered metal mesh. The Au mesh effectively doped the graphene layer to create a p-type electrode. Using Raman spectra, work function variations, and the transfer length method (TLM), we verified the effect of doping the graphene layer after depositing a very thin metal layer on the graphene layers. From our results, we suggest that the nature of the contact is an important criterion for improving the electrical and optical performance of hybrid TCLs, and the method of doping graphene layers provides new opportunities for solving contact issues in other semiconductor devices.

## Introduction

Graphene-based transparent conductive layers (TCLs) have been attracting attention as alternatives to conventional TCLs in optoelectronic devices, such as solar cells and light-emitting diodes (LEDs). They offer several advantages over existing oxide-based TCLs, including (i) high transparency without wavelength dependency, (ii) high conductivity, and iii) good flexibility^1, 2^. Graphene itself has a high value of sheet resistance (300–1500 Ω/□) resulting from imperfect growth and is therefore inappropriate for use in TCLs^3–5^. There have been multiple efforts to modify graphene using metal nanoparticles, metal nanowires, and a metal mesh to achieve the necessary performance characteristics and make graphene a suitable replacement for the oxide-based TCLs in UV LEDs and flexible devices^5–12^. However, despite reports of excellent performance for modified graphene, graphene/metal nanoparticle hybrid TCLs still exhibit unacceptable levels of sheet resistance caused by the discrete nanoparticles’ structures^6^. Moreover, even when the graphene/metal nanowire hybrid TCLs exhibit proper values for their sheet resistance (10–100 Ω/□) and transmittance (~85%), it has been difficult to control the uniformity of the thin nanowire films precisely and ensure their reliable contact characteristics in optoelectronic devices^5, 7, 12^. These drawbacks have led to the degradation of electrical contact properties in attempts to fabricate practical optoelectronic devices.

Among the various candidates, graphene/metal mesh hybrid TCLs are noteworthy for their use in practical applications because they have a low value of sheet resistance (1–100 Ω/□) and high transparency (~90%). Furthermore, the electrical and optical properties of hybrid TCLs can be sufficiently controlled by rationally designing the features of the metal mesh, such as the gaps between lines, line widths, and thicknesses.

Despite these advantages, there are presently few research results reporting the development of graphene/metal mesh hybrid TCLs for optoelectronic devices, and performance reported for both the electrical and optical properties of hybrid TCLs have fallen short of expectations^8–10^. We assume that the undesirable results for the hybrid TCLs in optoelectronic devices can be attributed to several issues: (i) the structure of the metal mesh, (ii) the optimum position of each material due to its hybridization, and (iii) the contact resistance of TCLs in devices.

We have previously reported the successful design of a metal mesh, and solved the structural issue in GaN-based near ultra-violet light-emitting diodes (NUV LEDs) by adding a supporting layer in the metal mesh to guarantee its stable contact properties^13^. However, achieving high-performance conditions for actual optoelectronic devices still requires determining the optimum position of each material and improving its contact properties with the p-GaN layer.

In this work, we demonstrate performance when a graphene layer is located either above or below the metal mesh, the doping of the graphene layer by incorporating a dual-layered metal mesh, and their effect on the performance of GaN-based NUV LEDs. Thanks to the high transparency of the hybrid films in the UV region, we were able to obtain remarkable optical properties in GaN-based NUV LEDs compared to conventional ITO. We used the metal mesh structure as reported in our previous work (150-um gap between metal lines, 5-um width for the metal line, and 150-nm thickness for the main metal line with 5-nm thickness for the very thin metal interlayers)^13^. We observed huge differences in the electrical and optical properties depending on the location of the graphene in the NUV LEDs even if each TCL showed similar sheet resistance and transmittance values, regardless of the graphene’s location. A main factor in these differences was the change made in the contact with the p-GaN layer, accomplished by changing the location of the graphene. Furthermore, we adopted a dual-layered metal mesh (an Ag mesh with a very thin metal mesh) to dope the graphene layer. Giovannetti *et al*. first reported the effect of doping a graphene layer by depositing metal, and several groups have since reported on the principles involved in the doping effects, both theoretically and experimentally^14, 15^. However, to date, there have been few results involving practical devices. In our work, we have obtained improvements for the contact properties in the NUV LEDs by doping the graphene layer with a 5 nm-thick Au/150 nm-thick Ag mesh, and we have verified the doping effect through Raman spectra, work function variations, and transfer length methods (TLMs). The superior changes in the graphene layer using this simple metal deposition technique provide many opportunities to control contact properties in other semiconductor devices.

## Results

**Figure 1:**
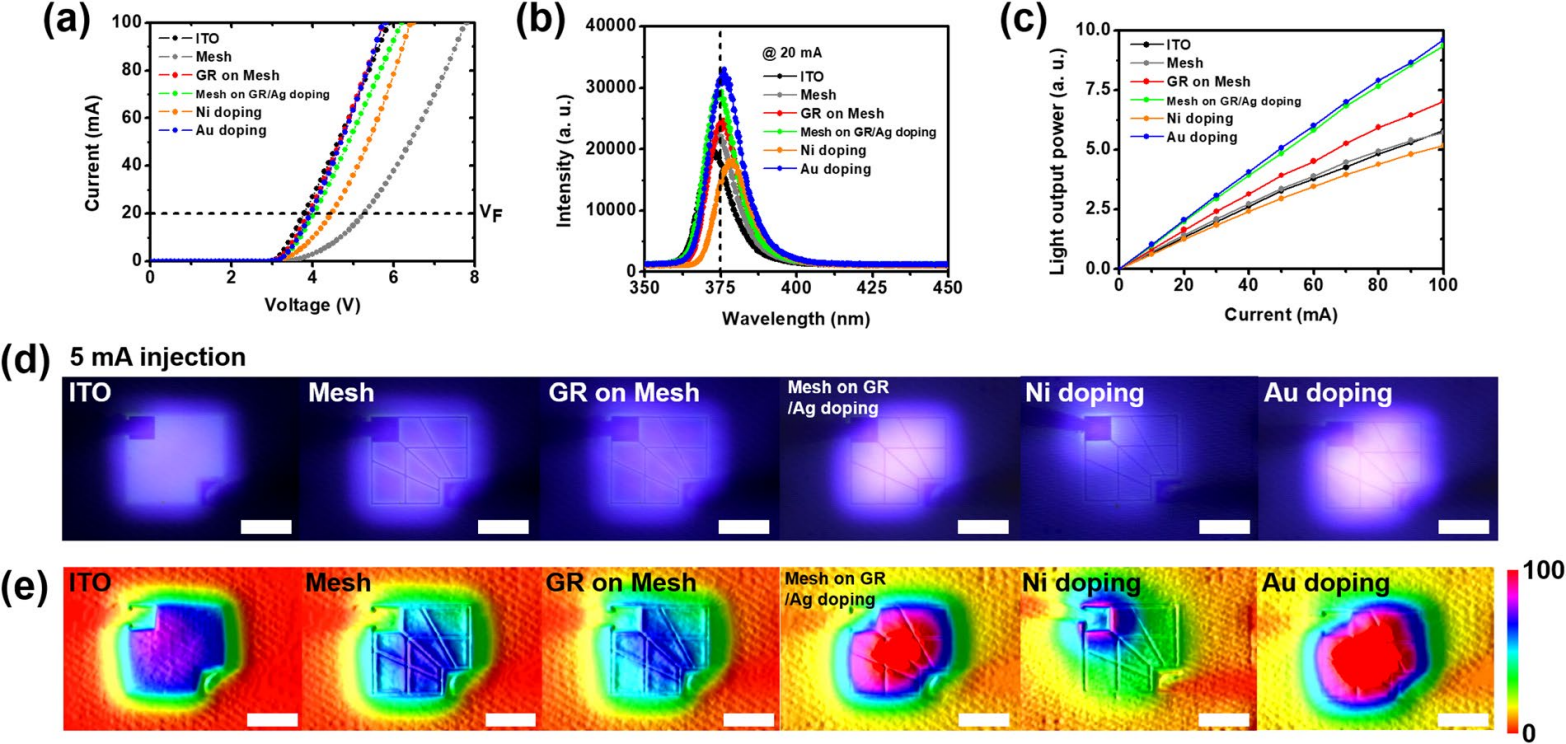
Electrical and optical properties of LED devices fabricated with each TCL: (**a**) current–voltage characteristics, (**b**) electroluminescence spectra at 20-mA, and (**c**) light output power versus input current. (**d**) Light emission images of LED devices at 5-mA. (**e**) Contour images of light intensity concerning (**d**).

Figure 1 shows the electrical and optical properties of NUV LEDs fabricated with the six types of TCL. We blindly measured ten different samples and used the average of the measured values in this work. We specified a range of −10–10 V for all NUV LEDs with current-voltage measurement limited to 100-mA. In addition, the range was set to 350–800 nm for electroluminescence measurement and the light output power measurement was performed by increasing from 0 mA to 100 mA with 10 mA steps based on the input current. All ranges shown in the figures were neatly organized for easy viewing. We were able to observe two remarkable factors and we can describe the results by separating them into two categories.

1. Even when the metal mesh and/or hybrid films exhibited similar sheet resistances and transmittance values, significant differences were observed in the electrical and optical performances of the NUV LEDs depending on the existence and location of the graphene (prime-location graphene).
2. We were able to dope the graphene layers by depositing a dual-layered metal mesh on them; this improved the contact resistance for the p-GaN layer (metal-doped graphene). Since NUV LEDs fabricated with a 150 nm-thick Ag mesh combined with graphene could act as both prime-location graphene and metal-doped graphene, we designated them “Mesh on GR/Ag doping”.

As can be seen in Fig. 1, we investigated the current-voltage characteristics, the electroluminescence spectra at 20-mA injection, and the light output power versus the input current. In the “Mesh”, “GR on Mesh”, and “Mesh on GR/Ag doping” samples, we were able to observe huge differences in the electrical and optical properties depending on the existence and location of the graphene layer. First, the NUV LEDs fabricated with “GR on Mesh” and “Mesh on GR/Ag doping” showed improved turn-on voltage values (3.9 V for “GR on Mesh” and 4.1 V for “Mesh on GR/Ag doping”) and series resistance (33.4 Ω for “GR on Mesh”, and 36.1 Ω for “Mesh on GR/Ag doping”) compared to “Mesh” (4.6 V for turn-on voltage and 51.8 Ω for series resistance). This improvement was attributed to covering the metal mesh with the graphene layer “above” the metal mesh in “GR on Mesh” and networking the metal mesh with the graphene layer “below” the metal mesh in “Mesh on GR/Ag doping”. Although we obtained enhancements in the electrical properties of “GR on Mesh” and “Mesh on GR/Ag doping”, there were slight differences depending on the location of the graphene layer. Further details of the cause are described in the discussion section. In addition to changing the graphene location in the metal mesh, we tested a dual-layered metal mesh on the graphene layer to obtain doping effect of a graphene layer. Surprisingly, we were able to observe stark differences in the values of the turn-on voltage (4.5 V for “Ni doping” and 4 V “Au doping”) and the series resistance (46.7 Ω for “Ni doping” and 33.9 Ω “Au doping”) depending on the type of very thin metal mesh in direct contact with the graphene layer. In particular, the NUV LEDs with “Au doping” even showed electrical properties comparable to those with “ITO” (3.7 V for the turn-on voltage and 25.4 Ω for the series resistance). We verified the doping effect of the graphene layer through Raman spectra, work function variations, and TLMs. Figure 1(b) shows the electroluminescence spectra of the NUV LEDs with the various TCLs at ~375 nm wavelength, which exhibited the maximum peak intensity for “Au doping.” As can be seen in Fig. 1(c), the enhanced performance in the light output power of the NUV LEDs was similar to the aforementioned electrical properties. Although the NUV LEDs with “Mesh” showed low light output power with the overconsumption of electrical power, the light output power of the NUV LEDs was improved by combining graphene (“GR on Mesh”) and graphene/Ag doping (“Mesh on GR/Ag doping”). The light output power of the NUV LEDs with “Mesh on GR/Ag doping” was much higher than that of the NUV LEDs with “GR on Mesh”. The differences originated from the use of different locations for the graphene layer with the metal mesh. This is discussed in greater detail in the discussion section.

Furthermore, the light output power of the NUV LEDs exhibited huge variations depending on the type of very thin metal mesh layer on the graphene layer. Specifically, while NUV LEDs with “Ni doping” exhibited the lowest light output power among all samples, we obtained the highest light output power in the NUV LEDs with “Au doping”. Figure 1(d) and (e) show the light emission images and the light intensity contour images extracted via image processing from Fig. 1(d) of each NUV LED at 5-mA injection. Based on the light emission and contour images, we were able to confirm that current crowding was somehow shown in the NUV LEDs with “ITO”. Moreover, we observed that the light emission of the NUV LEDs with “Mesh” was primarily focused on the side of the metal mesh. Although the light emission of the NUV LEDs with “GR on Mesh” seemed similar to that of the NUV LEDs with “Mesh”, the NUV LEDs with “GR on Mesh” showed better current spreading from the side of the metal mesh due to the graphene layer (see Figure S1 and the additional description for further information). The current crowding seemed higher in the NUV LEDs with “Mesh” compared to those with “GR on Mesh” was due to the overconsumption of electrical power in the NUV LEDs with “Mesh”, as can be seen in Fig. 1(a). The light emission of the NUV LEDs with “Mesh on GR/Ag doping” was more uniform and brighter than that of “GR on Mesh”; the reason for this is discussed further in the discussion section. Meanwhile, the NUV LEDs with “Au doping” showed both the most uniform and the strongest light emission. Thus, the main factor behind the significantly enhanced light output power in the NUV LEDs with “Au doping” was the even better current spreading caused by strongly reducing the sheet resistances with the Ag-mesh-combined graphene layer and improving current injection with the graphene doping effect for the 5 nm-thick Au layer designated as a dual-layered metal mesh. Therefore, we were able to obtain a greatly enhanced light output power from the NUV LEDs with “Au doping”, up to 165.2% greater than “ITO” with a 100-mA injection. The NUV LEDs with “Au doping” showed the best performance, considering both the electrical and optical properties.

## Discussion

### Prime-location grapheme

**Figure 2:**
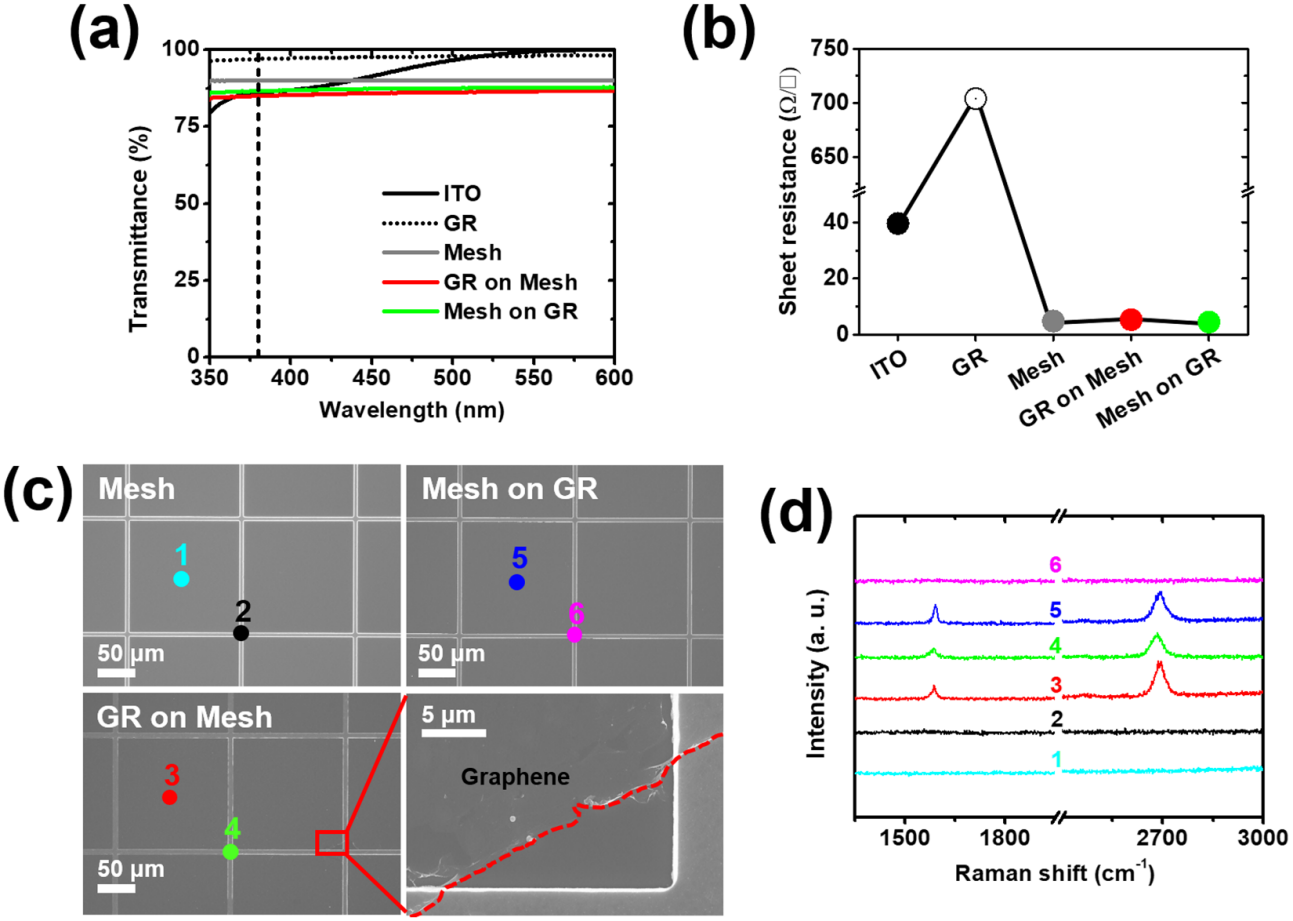
(**a**) Transmittance values for “ITO”, a graphene layer (“GR”), “Mesh”, “GR on Mesh”, and “Mesh on GR”. (**b**) Sheet resistance values with respect to each TCL formed on DSPS substrates. (**c**) SEM images of “Mesh”, “Mesh on GR”, and “GR on Mesh”. The magnified SEM image indicates the torn and rolled-up graphene layer in “GR on Mesh”. (**d**) Raman spectra obtained from colored points shown in (**b**).

**Figure 3:**
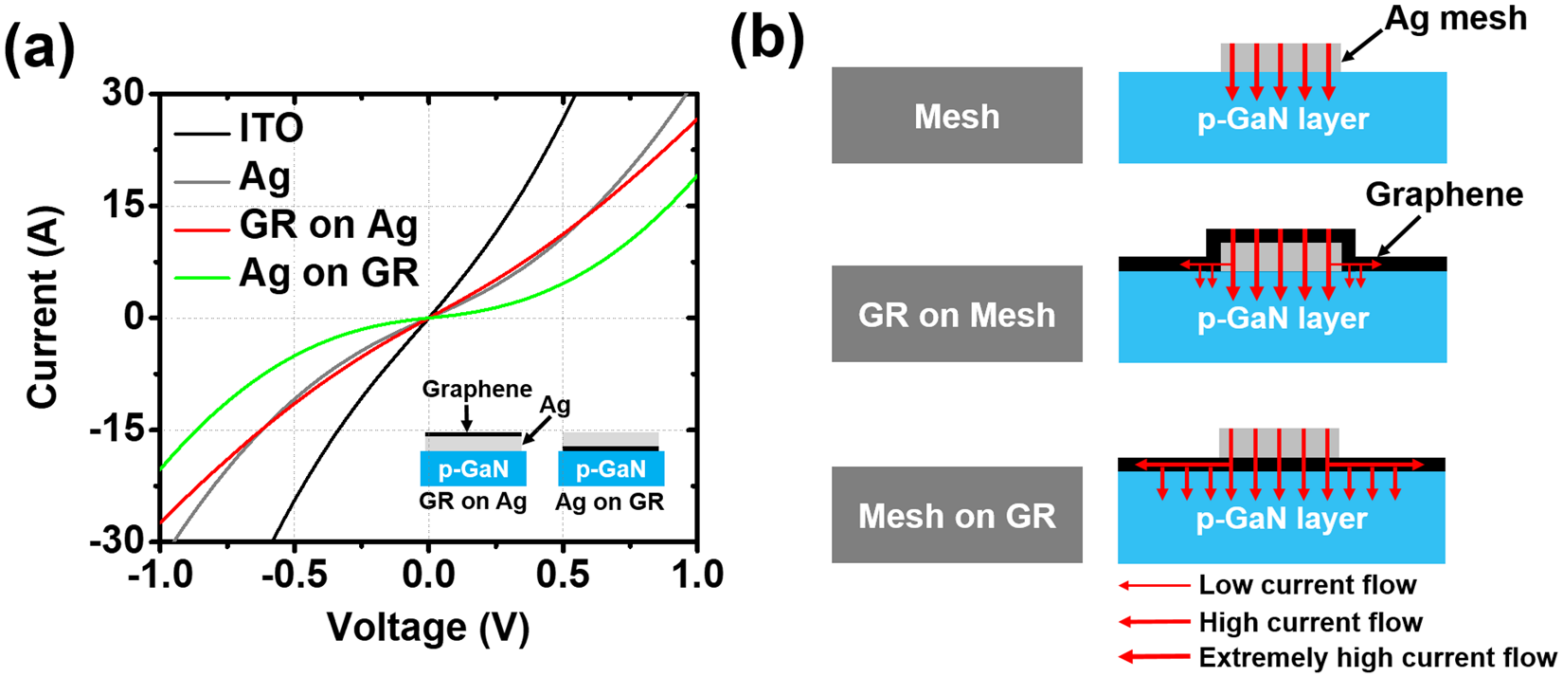
(**a**) Current–voltage characteristics extracted from TLM at 10-μm gap. The inset indicates TLM fabrication processes depending on the different locations of the graphene layer: a graphene layer on 150-nm-thick Ag (“GR on Ag”) and 150-nm-thick Ag on a graphene layer (“Ag on GR”). (**b**) The schematic illustration shows current flows depending on the existence and location of the graphene layer.

In order to obtain high-performance TCLs, we need to consider three important factors: the transparency values, sheet resistance values, and contact properties in the devices. We prepared a 150 nm-thick ITO (“ITO”), a graphene layer (“GR”), a 150 nm-thick Ag mesh (“Mesh”), a graphene layer on a 150 nm-thick Ag mesh (“GR on Mesh”), and a 150 nm-thick Ag mesh on a graphene layer (“Mesh on GR”) on double-side-polished sapphire (DSPS) substrates to investigate the transparency and sheet resistance. We measured the transmittance values in the range 350–600 nm with a UV-VIS-IR spectrometer (Cary 5000, Agilent Technologies), as shown in Fig. 2(a). The transmittance value of the ITO was 99.4% at 550 nm (visible wavelength) but was severely decreased to 85.9% at 375 nm (NUV wavelength) due to the absorption of the ITO layer. In contrast, transmittance values were constant regardless of the wavelength changes for the pristine graphene layer and Ag-mesh-combined graphene layers: 98.1% for “GR”, 92.2% for “Mesh”, 88.1% for “GR on Mesh”, and 89.3% for “Mesh on GR” at 550 nm and 96.9% for “GR”, 92.6% for “Mesh”, 87.1% for “GR on Mesh”, and 88.4% for “Mesh on GR” at 375 nm. The transmittance values in all TCLs incorporating the pristine graphene layer and Ag mesh were reduced by the same amount as the sum of the transmittance loss from the pristine graphene layer (~3%) and Ag mesh (~8%). We also measured sheet resistance values, one of the electrical properties important for current spreading in TCLs as can be seen in Fig. 2(b). High sheet resistance values can lead to severe current crowding in UV LEDs due to the resistive p-GaN layers^16^. Even though the sheet resistance values of the “ITO” and “GR” TCLs were high (39.6 Ω/□ and 704 Ω/□, respectively), those of the other TCLs with the Ag mesh structures were very low: 4.88 Ω/□ for “Mesh”, 5.39 Ω/□ for “GR on Mesh”, and 4.54 Ω/□ for “Mesh on GR”. We were able to observe differences in the sheet resistance values based on the existence and location of the graphene layer. The lower sheet resistance values of “Mesh on GR” than “Mesh” were attributed to the graphene layer bridging the gaps of the Ag mesh. However, the higher sheet resistance values of “GR on Mesh” than “Mesh” were due mostly to the torn and rolled-up condition of the graphene layers, even though the graphene layers connected the Ag mesh, as shown in Fig. 2(c). We observed the “Mesh” and Ag-mesh-combined graphene layers (“Mesh on GR” and “GR on Mesh”) using SEM images (FESEM, Hitachi S-4700). Although there were no differences in the surface images of all TCLs at low magnification, we were able to identify torn and rolled-up graphene layers at the intersections of the Ag mesh lines for “GR on Mesh” at high magnification. In fact, when the graphene layers were transferred onto a smooth surface, they were well laid out, and we were able to distinguish the graphene layers located above/below the Ag mesh (see Figure S2). The flaws generated when transferring the very thin graphene layer onto highly irregular mesh structures (particularly the intersections) degraded the transparency and sheet resistance in “GR on Mesh”. Similar problems have been observed with sparse nanorod structures^17^. Next, we investigated the Raman spectra (Renishaw, 514 nm-line of an Ar-ion laser) to identify the graphene layers above/below the Ag mesh. In Fig. 2(d), the colored lines are the results of the Raman spectra obtained from the colored regions in the SEM images in Fig. 2(c). The “1” and “2” represent the Raman spectra on a gap region and an Ag mesh region, respectively, and there is no G peak or 2D peak. The “3” and “4” in the “GR on Mesh” indicate G peaks (1587 cm^−1^ and 1586 cm^−1^) and 2D peaks (2694 cm^−1^ and 2683 cm^−1^), respectively. Notably, there were peak shifts in the G and 2D peaks in “4” on the graphene layer on the Ag mesh. These shifts are further discussed in the section on metal-doped graphene. In contrast, the G peak (1591 cm^−1^) and 2D peak (2693 cm^−1^) appeared only in a gap region in the “Mesh on GR” TCL. To investigate the contact properties between the TCLs and p-GaN layers depending on the location of the graphene layers, we fabricated TLM samples using pure p-GaN layers. The process of preparing the TLM samples is shown in the inset of Fig. 3(a). We fabricated the TLM samples using a planar Ag layer rather than an Ag mesh due to the small available area of contact in the mesh types. We measured the resistance values of four TLM samples by probing with two electrodes while increasing the distance between the two electrodes (see Figure S3). The resistance values increased when the probe interval increased in all samples. From the measured resistance values, we were able to obtain specific contact resistance values: 0.14 Ω·cm^2^ for “ITO”, 0.53 Ω·cm^2^ for “Ag”, 0.81 Ω·cm^2^ for “GR on Ag”, and 1.23 Ω·cm^2^ for “Ag on GR” (see Table S1). Of all the samples, “ITO” exhibited the lowest specific contact resistance value. This is attributed to the higher work function of “ITO” (4.7 eV for ITO, 4.5 eV for Ag, and 4.49 eV for graphene (see Figure S4), making it closer to that of p-GaN and better contact properties achieved through thermal annealing at 600 °C^18, 19^. Since the same materials, Ag layers, are in contact with the p-GaN in both “Ag” and “GR on Ag”, their specific contact resistance values are similar. However, the specific resistance of “Ag on GR” was higher than those of the others. We were able to determine that the ranking of contact characteristics was “ITO” > “Ag” > “GR on Ag” > “Ag on GR” as can be seen in Fig. 3(a). Based on the contact properties from the specific resistance and current spreading properties from the light emission uniformity, we obtained a current flow model, shown in Fig. 3(b). With the Ag mesh, the current locally flows from the mesh to the p-GaN layer due to the inherently high resistance of the p-GaN layer, which causes degradation of the electrical and optical properties. Furthermore, because the Ag mesh has a slightly higher work function than the graphene layer directly in contact with the p-GaN layer in “GR on Mesh”, almost the entire current flows through the Ag mesh, though a small amount of current flows through the graphene layer. However, in “Mesh on GR”, the current uniformly flows through the entire area of the p-GaN layer due to the direct and complete contact of the graphene layer with the p-GaN layer. As a result, despite the similar sheet resistances and transmittances for “Mesh”, “GR on Mesh”, and “Mesh on GR”, the NUV LEDs with those TCLs differed significantly in performance due to their different contact characteristics, which were determined by the existence and location of the graphene layer. When the p-GaN layer was directly connected to the graphene layer rather than Ag, as with “Mesh on GR”, the NUV LEDs showed better performance.

### Metal-doped grapheme

Even though the remarkable transparency and sheet resistance of the TCLs was established, some uncertainty remains regarding device performance. For example, we previously observed that there were huge variations depending on the location of the graphene layer, even when they had similar transparency and sheet resistance values. Accordingly, the contact issues require careful consideration to obtain high-performance TCLs. To improve contact properties when using graphene-based TCLs, the graphene is typically doped using chemical methods^20, 21^. However, the chemical doping of graphene has several disadvantages, such as instability in external environments. For that reason, in this work we doped the graphene by simply depositing a very thin metal layer. A number of researchers have previously reported the potential doping of graphene using thin metal layers^14, 15, 22^. By investigating with Raman analysis, the measurement of work function, and TLMs, we determined the effect of doping the graphene using very thin metal deposition, and we describe how to incorporate their advantages in our devices below.

#### Raman spectra

**Figure 4:**
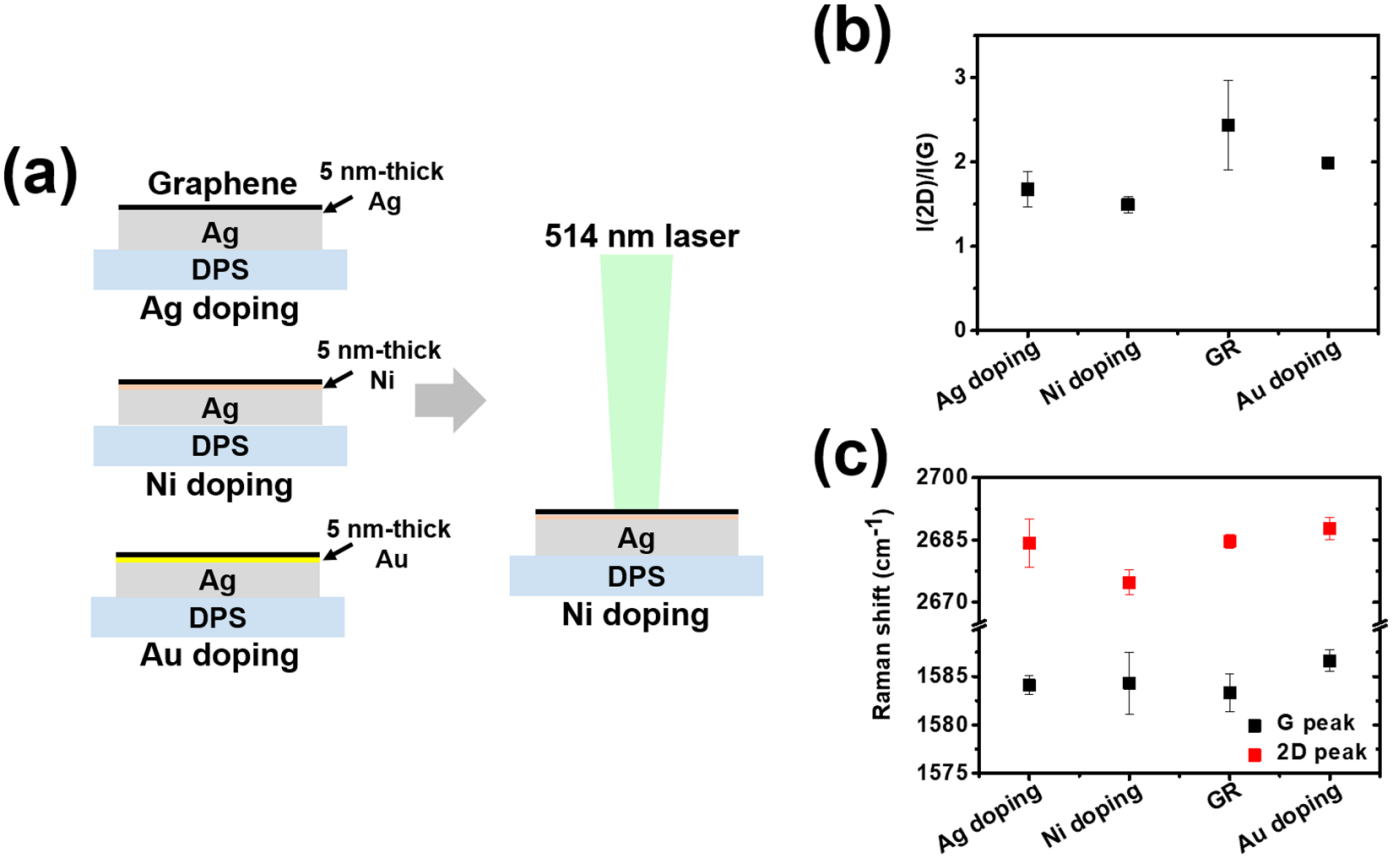
(**a**) Schematic illustration shows sample preparations for Raman analysis of each metal-doped graphene layer. (**b**) and (**c**) represent statistical intensity ratio I(2D)/I(G) and shifts for the G peak and 2D peak obtained from Raman spectra, respectively.

Raman spectroscopy is one of the most powerful tools for investigating changes in the graphene layer. Many research groups have used Raman spectroscopy to characterize chemically doped graphene^23, 24^. In order to verify the doping effect of the very thin metal layer on the graphene layer, we prepared samples for Raman analysis, as can be seen in Fig. 4(a). We measured Raman spectra at ten different spots for each of the graphene layers. First, we investigated the ratio of intensity of the 2D peak [I(2D)] and G peak [I(G)] for “Ag doping”, “Ni doping”, and “Au doping”, as can be seen in Fig. 4(b). The graphene layers for “Ag doping”, “Ni doping”, and “Au doping” showed a decrease of I(2D)/I(G) compared to the pristine graphene layer with either n-type doping or p-type doping, depending on the doping effect. In previous studies, graphene layers with Ag and Ni layers produced n-type doping, while those with Au layers produced p-type doping. Shifts in the G peak and 2D peak are commonly used to distinguish the type of doping of the graphene layer, and the 2D peak predominantly determines the type of doping (downshifting for n-type doping, upshifting for p-type doping). As can be seen in Fig. 4(c), we observed upshifting for both the G peak and 2D peak in “Au doping”, although there was slight upshifting for the G peak and downshifting for the 2D peak in “Ag doping” and “Ni doping”. Thus, we were able to assume that “Ag doping” and “Ni doping” were n-type doping and that “Au doping” was p-type doping. In particular, “Ni doping” showed severe downshifting, which typically reflects strong n-type doping.

#### Work function

**Figure 5:**
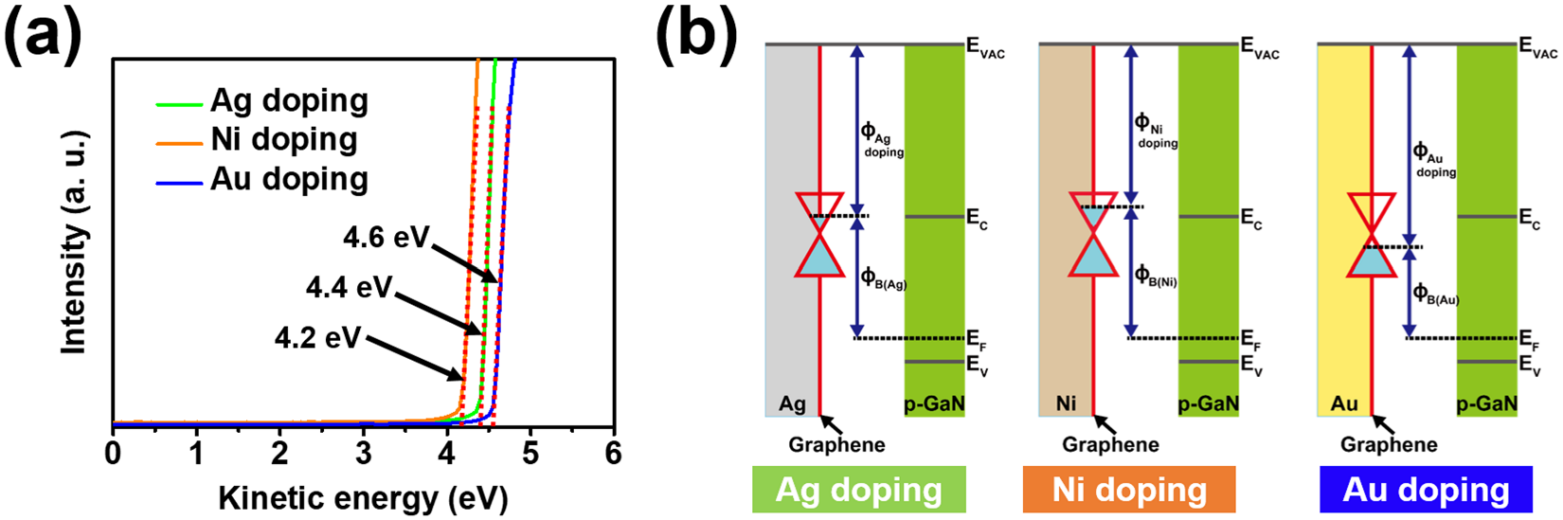
(**a**) Results of work function measured by ultraviolet photoelectron spectroscopy with respect to metal-doped graphene layers. (**b**) Energy band diagrams between metal-doped graphene layers and p-GaN layers.

In addition to using Raman spectra to verify doping type, we checked the doping effect of graphene by measuring the work function of the graphene with the very thin metal layer using ultraviolet photoelectron spectroscopy. Figure 5(a) shows the results of the work function with respect to “Ag doping” (4.4 eV), “Ni doping” (4.2 eV), and “Au doping” (4.6 eV). The work function is defined as the difference in value between the vacuum level and the Fermi energy level of the materials. Thus, a work function lower than that of the pristine graphene layer (4.49 eV) means that the graphene layer has been modified with n-type doping, while a higher work function indicates p-type doping. In this way, we were able to double-check the type of doping for “Ag doping” (n-type doping), “Ni doping” (n-type doping), and “Au doping” (p-type doping). Moreover, as we had previously observed in the Raman analysis, the graphene with “Ni doping” exhibited strong n-type doping because of the severe decrease in the work function. In general, for “Ni doping” and “Au doping”, the work function of the graphene layer should increase as p-type doping by charge transfer from the graphene layer to the metal layers by differences of potential energy. However, there is different charge transfer in “Ni doping”. In “Ni doping”, the graphene layer interacts strongly with the Ni layer due to small equilibrium interfacial distance, hybridizing the graphene *p*_*z*_ states with metal *d* states^24^. This overlap of the band structure results in electron diffusion from the Ni layer to the graphene layer by repulsive force of the electrons caused by Pauli exclusion principle and causes “Ni doping” n-type doping. Thus, there are two types of adsorption for metal-graphene: physisorption (by large equilibrium interfacial distance) and chemisorption (by small equilibrium interfacial distance). It is necessary to use materials with large work functions for the p-GaN layer to form better contact properties. In Fig. 5(b), we show the energy band diagrams of the metal-doped graphene layers and p-GaN layers based on the work function values for “Ag doping”, “Ni doping”, and “Au doping”. The graphene layers with “Ag doping” and “Ni doping” increase the barrier height that holes need to overcome between the graphene and p-GaN layers. Specifically, it is almost impossible to obtain good contact properties with the graphene layer with “Ni doping” due to the large barrier height. In contrast, the graphene layers with “Au doping” reduce the barrier height between the graphene and p-GaN layers, thus reducing the contact resistance.

#### TLM samples

**Figure 6:**
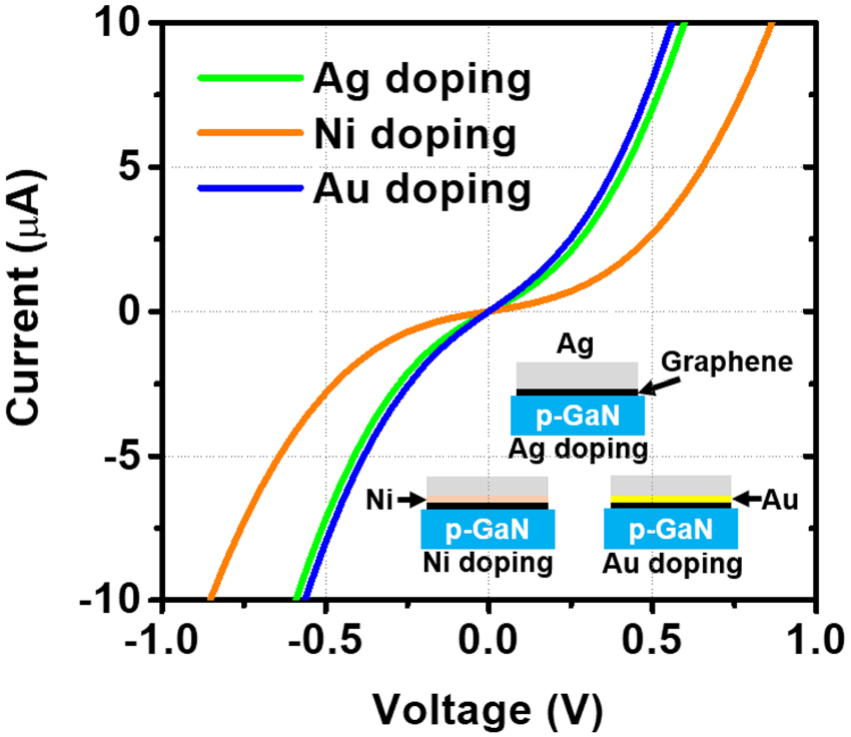
Current–voltage characteristics extracted from TLM at 10-μm gap. The inset indicates the preparation of TLM samples for each metal-doped graphene layer: 150-nm-thick Ag deposited on a graphene layer (“Ag doping”), 150-nm-thick Ag/5-nm-thick Ni deposited on a graphene layer (“Ni doping”), and 150-nm-thick Ag/5-nm-thick Au deposited on a graphene layer (“Au doping”).

Finally, we fabricated TLM samples for each metal-doped graphene layer to investigate the contact properties with the p-GaN layers. The preparation of TLM samples is shown in the inset of Fig. 6. To ensure a fair comparison, we deposited equally thick (i.e., 150 nm-thick) Ag layers on different thin metal layers (5-nm-thick Ag layer, 5-nm-thick Ni layer, and 5-nm-thick Au layer). Furthermore, we fabricated the TLM samples without additional contact pads (30-nm-thick Cr/300-nm-thick Au) to observe their pristine contact characteristics. We measured the resistance values for ten different samples of “Ag doping”, “Ni doping”, and “Au doping” and created a graph of the average values (see Figure S5). From the measured resistance values, we were able to obtain the specific contact resistance values: 0.39 Ω·cm^2^ for “Ag doping”, 1.1 Ω·cm^2^ for “Ni doping”, and 0.08 Ω·cm^2^ for “Au doping” (see Table S2). It is worth noting that the specific contact resistance values were dramatically changed simply by adding different types of very thin metal layers between the thick Ag layers and the graphene layer. The specific contact resistance values for “Au doping” were sharply improved compared to those of “Ag doping” and “Ni doping”. Although the graphene layer with the Ni interlayer showed severe degradation in the I-V characteristics, the graphene layers with the Au interlayer indicated much better as can be seen in Fig. 6. We were able to achieve the best contact properties on the p-GaN layer with the graphene layer with “Au doping”. In addition, we measured transmittance and sheet resistance values for each TCL to investigate the effect of the additionally deposited thin metal mesh (see Figure S6). We were able to confirm that there were no significant changes in the transmittance and sheet resistance. As a result, the high performance of the NUV LEDs with “Au doping” was attributed to the reduction in barrier height between the graphene and the p-GaN layer through p-type doping of the graphene layer while maintaining low sheet resistance and moderate transmittance.

## Conclusion

In conclusion, we demonstrated high-performance graphene/metal mesh TCLs after determining the prime location for the graphene and performing p-type doping of the graphene in the NUV LEDs. For better physical stability and current spreading, the graphene should be located below the metal mesh and directly in contact with the p-GaN layer. Furthermore, although the NUV LEDs with “Mesh on GR/Ag doping” showed outstanding optical properties, there was a tradeoff with the electrical properties due to the high contact resistance. Through Raman analysis, the measurement of work function, and TLMs, we were able to confirm that graphene can be doped with Ni and Ag for n-type doping and with Au for p-type doping. To further reduce the high contact resistance of “Mesh on GR/Ag doping”, we adopted a dual-layered metal mesh by inserting a 5-nm-thick Au mesh between the Ag mesh to produce the p-type doping of graphene. As a result, we were able to obtain high-performance NUV LEDs with “Au doping”, leading to an up to ~165% improvement compared to conventional ITO TCLs. Our results show that the contact properties in the devices are as critical as the transparency and sheet resistance of the materials and that the contact properties in the devices can be tuned by doping the graphene with metal deposition. In particular, using the simple metal deposition method to dope graphene offers news opportunities to solve contact issues in other semiconductor devices.

## Methods

### Fabrication of LEDs with a variety of TCLs

**Figure 7:**
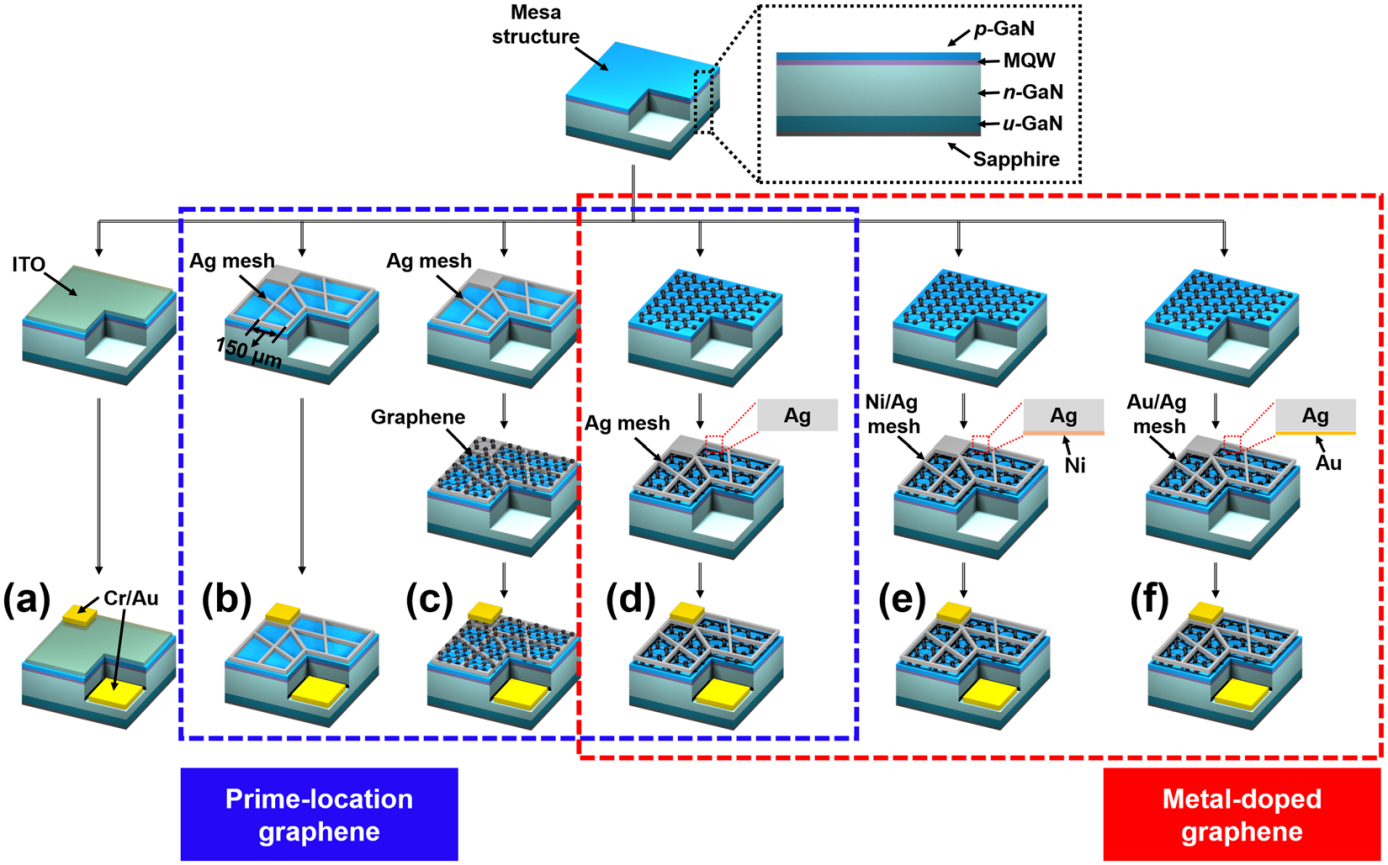
Schematic illustration showing fabrication process of LED devices, using six types of TCLs: (**a**) 150-nm-thick ITO (“ITO”), (**b**) 150-nm-thick Ag mesh (“Mesh”), (**c**) a graphene layer on 150-nm-thick Ag mesh (“GR on Mesh”), (**d**) 150-nm-thick Ag mesh on a graphene layer (“Mesh on GR/Ag doping”), (**e**) 150-nm-thick Ag mesh and 5-nm-thick Ni mesh on a graphene layer (“Ni doping”), and (**f**) 150-nm-thick Ag mesh and 5 nm-thick Au mesh on a graphene layer (“Au doping”).

Figure 7 shows the process for fabricating LED devices with various TCLs. By using metal organic chemical vapor deposition (MOCVD), 375-nm NUV LEDs were grown. In order to form mesa structures, 300-nm-thick SiO_2_ layers were prepared by plasma-enhanced chemical vapor deposition (PECVD) for mesa masks. The mesa structures were formed through photolithography with positive photoresist (PR) (AZ 4330) and inductively coupled plasma (ICP) etching, as can be seen in Fig. 7. Then, we stripped the PR and SiO_2_ layers using acetone and an HF solution sequentially. Using the mesa structures of the NUV LEDs, we then fabricated six types of TCLs on the p-GaN layers: 150-nm-thick ITO (“ITO”), 150-nm-thick Ag mesh (“Mesh”), a graphene layer on 150-nm-thick Ag mesh (“GR on Mesh”), 150-nm-thick Ag mesh on a graphene layer (“Mesh on GR/Ag doping”), 150-nm-thick Ag mesh and 5 nm-thick Ni mesh on a graphene layer (“Ni doping”), and 150-nm-thick Ag mesh and 5-nm-thick Au mesh on a graphene layer (“Au doping”). For “ITO” samples, we made patterns using negative PR (AZ 2035) and deposited the 150-nm-thick ITO layers on the patterned p-GaN layers by e-beam evaporation (E-beam). After the PR lift-off, the ITO layers were annealed by rapid thermal annealing at 600 °C for 5 minutes under ambient air. We subsequently completed the metal mesh in all of the TCLs (“Mesh”, “GR on Mesh”, “Mesh on GR/Ag doping”, “Ni doping”, and “Au doping”) by metal deposition process and graphene transfer process, as can be seen in Fig. 7. Finally, we simultaneously deposited Cr (30 nm)/Au (300 nm) layers on all the samples as the p- and n-metal pads and lifted off the PR.

### Preparation of TLM samples on p-GaN layers

We grew two TLM samples with a structure of 1-µm-thick pure p-GaN layers/2-µm-thick un-doped GaN layers on sapphire substrates. We measured the hole concentrations of the pure p-GaN layers using Hall measurements (model: HL 5500 supplied by BIO-RAD) and obtained values of 3.7 × 10^17^/cm^3^ and 7 × 10^17^/cm^3^ for the two samples. We employed lower hole concentration for investigating the different locations of the graphene and higher hole concentration for verifying the doping of the graphene in the TLM samples.

### TLM samples for prime-location grapheme

We deposited 300-nm-thick SiO_2_ on the pure GaN layers to make mesa masks using PECVD and made mesa patterns on the SiO_2_ layers using photolithography with AZ 4330. We formed the mesa structures by ICP etching and stripping out the PR. We removed the rest of the SiO_2_ layers with HF solution. Using the mesa structures, we then fabricated TLM samples with 100-μm width and 200-μm length with a range of gaps from 5-μm to 40-μm with a 5-μm interval. We prepared ITO and Ag samples for the TLM samples (“ITO” and “Ag”) using the same methods described in method section 1.

In order to verify how the contact properties differed depending on the graphene locations, we made two different types of TLM samples: one with a graphene layer on 150-nm-thick Ag (“GR on Ag”) and one with 150-nm-thick Ag on a graphene layer (“Ag on GR”). For “GR on Ag”, we deposited a 150-nm-thick Ag layer on the pure p-GaN layer after creating the TLM patterns and then transferred a graphene layer onto the Ag layer. For “Ag on GR”, we transferred a graphene layer onto the mesa structures of the pure p-GaN layers and deposited a 150-nm-thick Ag layer on the graphene layer. We completed the TLM samples by isolating the graphene layers using reactive ion etching (RIE) with TLM PR mask patterns and removing the rest of the PR.

### TLM samples for metal-doped grapheme

Using the same methods introduced in Sec. 2. 2-1, we made mesa structures on the pure p-GaN layers. To verify how doping the graphene affected the contact properties through thin metal interlayers, we prepared three types of thin metal interlayer on the graphene layer: 150-nm-thick Ag/5-nm thick Ag/graphene (“Ag doping”), 150-nm-thick Ag/5-nm-thick Ni/graphene (“Ni doping”), and 150-nm-thick Ag/5-nm-thick Au/graphene (“Au doping”).

For all TLM samples, we transferred the graphene layers onto the mesa structures of the pure p-GaN layers and deposited the thin metal layers by E-beam. Furthermore, 150-nm-thick Ag layers were equally deposited on the thin metal layers. The graphene layers were separated by RIE etching with TLM PR mask patterns, and the rest of the PR was removed.

### Preparation for Raman analysis and measurement of work function

To investigate the doping effect of graphene based on Raman shift, we prepared three types of graphene layers located on different thin metal layers, graphene on 5-nm-thick Ag (“Ag doping”), graphene on 5-nm-thick Ni (“Ni doping”), and graphene on 5-nm-thick Au (“Au doping”), as shown in Fig. 4(a). Based on depositing 150-nm-thick Ag layers on DSPS substrates, we additionally deposited thin metal layers on the Ag layers. Then, we transferred the graphene layers onto the thin metal layers.

## Electronic supplementary material

Supplementary Information

## References

[1] Bae S (2010). Roll-to-roll production of 30-inch graphene films for transparent electrodes. Nat. Nanotechnol..

[2] Pang S, Hernandez Y, Feng X, Müllen K (2011). Graphene as transparent electrode material for organic electronics. Adv. Mater..

[3] Jo G (2010). Large-scale patterned multi-layer graphene films as transparent conducting electrodes for GaN light-emitting diodes. Nanotechnology.

[4] Han T-H (2012). Extremely efficient flexible organic light-emitting diodes with modified graphene anode. Nat. Photonics.

[5] Seo TH (2015). Improving the graphene electrode performance in ultra-violet light emitting diode using silver nanowire networks. Opt. Mater. Express.

[6] Shim J-P (2013). Thin Ni film on graphene current spreading layer for GaN-based blue and ultra-violet light-emitting diodes. Appl. Phys. Lett..

[7] Li Z (2013). Enhanced performance of GaN-based light-emitting diodes with graphene/Ag nanowires hybrid films. AIP Adv..

[8] Kahng YH (2014). Highly conductive flexible transparent electrodes fabricated by combining graphene films and inkjet-printed silver grids. Sol. Energy Mater. Sol. Cells.

[9] Dong P (2014). Graphene on metal grids as the transparent conductive material for dye sensitized solar cell. J. Phys. Chem. C.

[10] Cha MJ, Kim SM, Kang SJ, Seo JH, Walker B (2015). Improved performance in flexible organic solar cells via optimization of highly transparent silver gird/graphene electrodes. RSC Adv..

[11] Gao T (2015). Hierarchical graphene/metal grid structures for stable, flexible transparent conductors. ACS Nano.

[12] Hwang B, Shin H-A-S, Kim T, Joo Y-C, Han SM (2014). Highly reliable Ag nanowire flexible transparent electrode with mechanically welded junctions. Small.

[13] Min J-H (2015). Ag-mesh-combined graphene for an indium-free current spreading layer in near-ultraviolet light-emitting diodes. RSC Adv..

[14] Giovannetti G (2008). Doping graphene with metal contacts. Phys. Rev. Lett..

[15] Khomyakov PA (2009). First-principles study of the interaction and charge transfer between graphene and metals. Phys. Rev. B.

[16] Min J-H (2017). Effect of *p*-GaN hole concentration on the stabilization and performance of a graphene current spreading layer in near-ultraviolet light-emitting diodes. Curr. Appl. Phys..

[17] Shim J-P (2016). Size-controlled InGaN/GaN nanorod LEDs with an ITO/graphene transparent layer. Nanotechnology.

[18] Margalith T (1999). Indium tin oxide contacts to gallium nitride optoelectronic devices. Appl. Phys. Lett..

[19] Kim DW, Sung YJ, Park JW, Yeom GY (2001). A study of transparent indium tin oxide (ITO) contact to p-GaN. Thin Solid Films.

[20] Güneş F (2010). Lyaer-by-layer doping of few-layer graphene film. ACS Nano.

[21] Liu H, Liu Y, Zhu D (2011). Chemical doping of graphene. J. Mater. Chem..

[22] Varykhalov A, Scholz MR, Kim TK, Rader O (2010). Effect of noble-metal contacts on doping and band gap of graphene. Phys. Rev. B.

[23] Dong X (2009). Doping single-layer graphene with aromatic molecules. Small.

[24] Gong C (2010). First-principles study of metal-graphene interfaces. J. Appl. Phys..

