# Supplementary material for: High-performance metal mesh/graphene hybrid films using prime-location and metal-doped graphene
Source: Sci Rep. 2017 Aug 31;7:10225. doi: 10.1038/s41598-017-10355-5 (PMC5579003; doi:10.1038/s41598-017-10355-5)
Supplement: Supplementary file 1 — Supplementary Information [file 41598_2017_10355_MOESM1_ESM.pdf]

Supplementary information for

High-performance graphene/metal mesh hybrid films through prime-location and metal-doped graphene

Jung-Hong Min,[1,2] Woo-Lim Jeong, [1,2] Hoe-Min Kwak, [1,2] and Dong-Seon Lee[1,2,]*

[1] School of Electrical Engineering and Computer Science, Gwangju Institute of Science and Technology, Gwangju 61005, South Korea

[2] Research Institute for Solar and Sustainable Energies, Gwangju 61005, South Korea

* **Corresponding author**. Tel.: +82 62 7152248; fax: +82 62 7152204

E-mail: dslee66@gist.ac.kr (D. S. Lee)

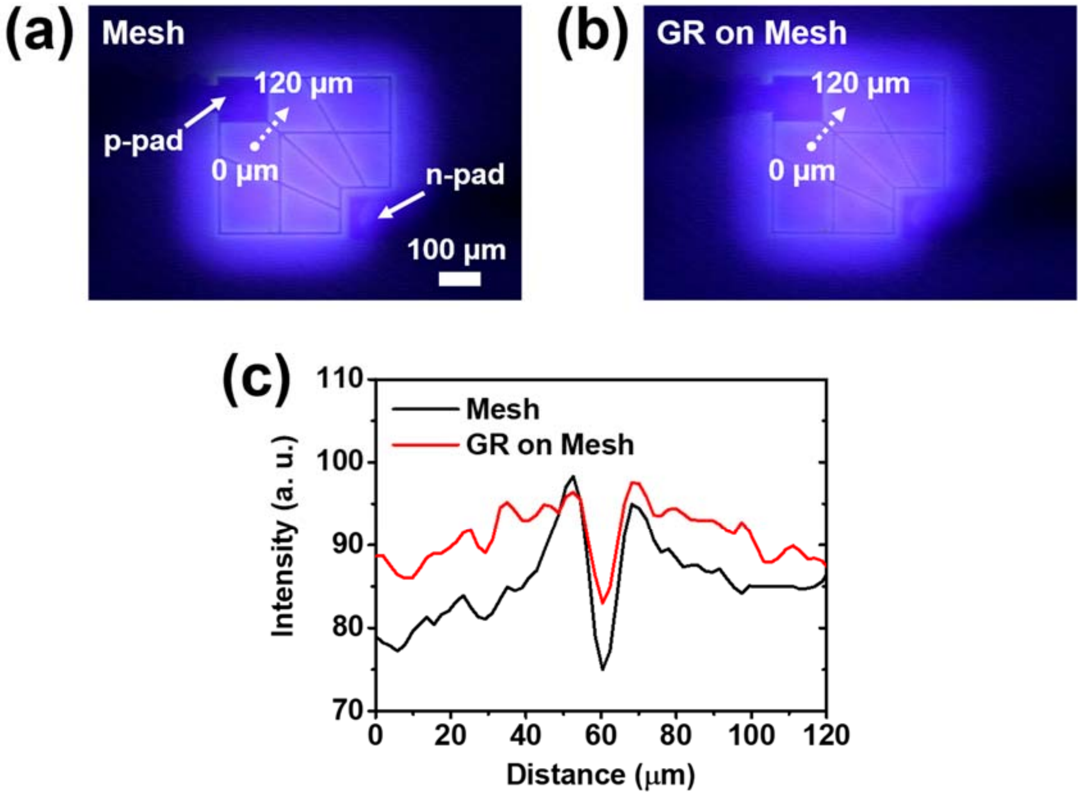

Figure S1. Light emission images of NUV LEDs: (a) "Mesh" and (b) "GR on Mesh". (c) Sectional light intensity versus the distance shown in (a) and (b).

In order to identify how much the light intensity differs depending on the graphene layer, we investigated the local light emission images of NUV LEDs with "Mesh" and "GR on Mesh," as shown in Figure S1(a) and (b). We firstly selected the local regions in the NUV LEDs, as indicated as white-dotted arrows, set start points as 0 μm and end points as 120 μm, and obtained the light intensity versus the distance from the start points to the end points by image processing using MATLAB software. As can be seen in Figure S1(c), we were able to observe V-shaped dim areas along the metal mesh. Furthermore, although the light intensity in "Mesh" was greatly reduced from the V-shaped dim area, that in "GR on Mesh" showed as more uniform, even away from the V-shaped dim area.

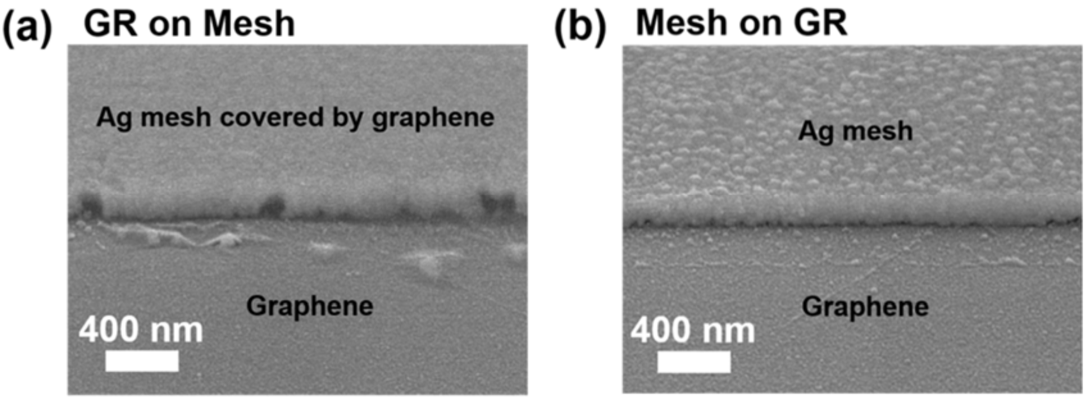

Figure S2. SEM images depending on the different locations of a graphene layer: (a) a graphene layer on 150-nm-thick Ag mesh ("GR on Mesh") and (b) 150-nm-thick Ag mesh on a graphene layer ("Mesh on GR").

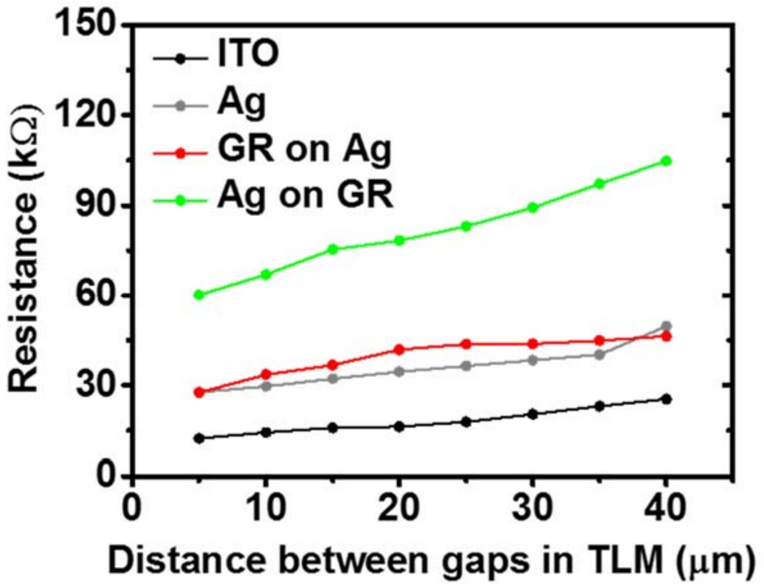

Figure S3. Resistance values obtained through transfer length method (TLM) for "ITO", "Ag", and depending on the different locations of a graphene layer.

Table S1. Specific contact resistances and contact resistances for "ITO", "Ag", and depending on the different locations of a graphene layer.

Types	Specific contact resistance ($\Omega\cdot cm^2$)	Contact resistance (kΩ)
**ITO**	0.14	5.1
**Ag**	0.53	12
**GR on Ag**	0.81	14.3
**Ag on GR**	1.23	27.3

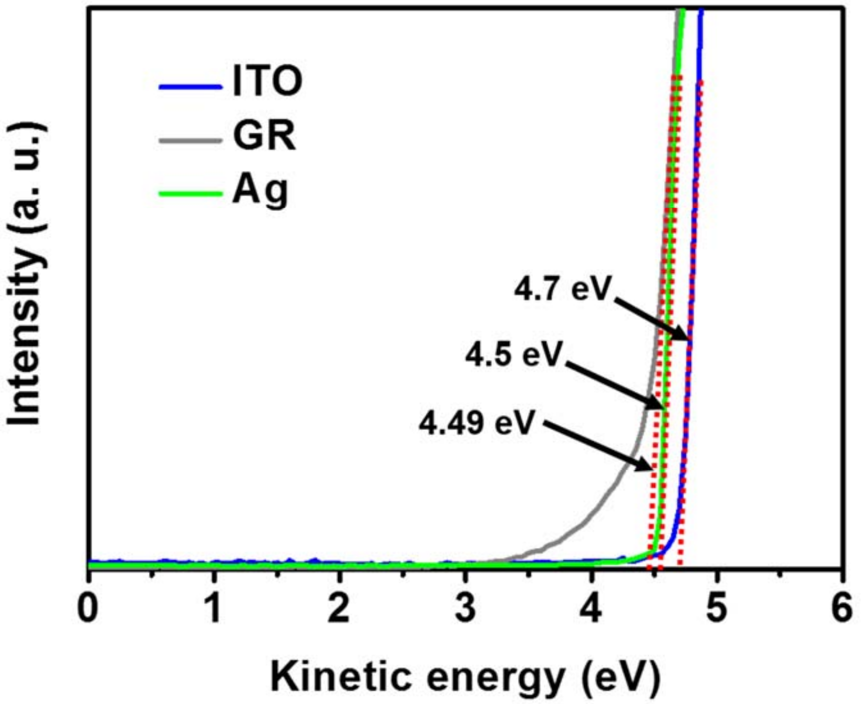

Figure S4. Results of work function measured by ultraviolet photoelectron spectroscopy for a 150-nm-thick ITO layer (“ITO”), a graphene layer (“GR”), and a 150-nm-thick Ag layer (“Ag”), respectively.

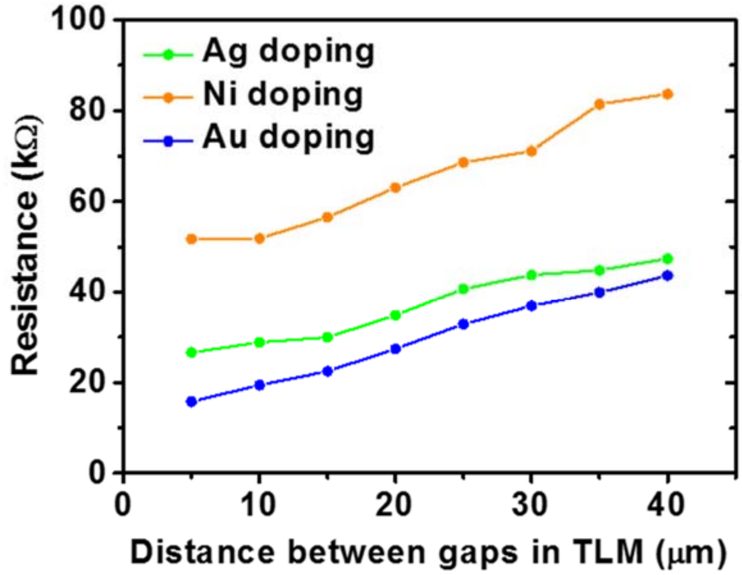

Figure S5. Resistance values obtained through transfer length method (TLM) for metal-doped graphene.

Table S2. Specific contact resistance and contact resistance values for metal-doped graphene.

Types	Specific contact resistance ($\Omega \cdot cm^2$)	Contact resistance (kΩ)
**Ag doping**	0.39	11.3
**Ni doping**	1.1	21.3
**Au doping**	0.08	5.6

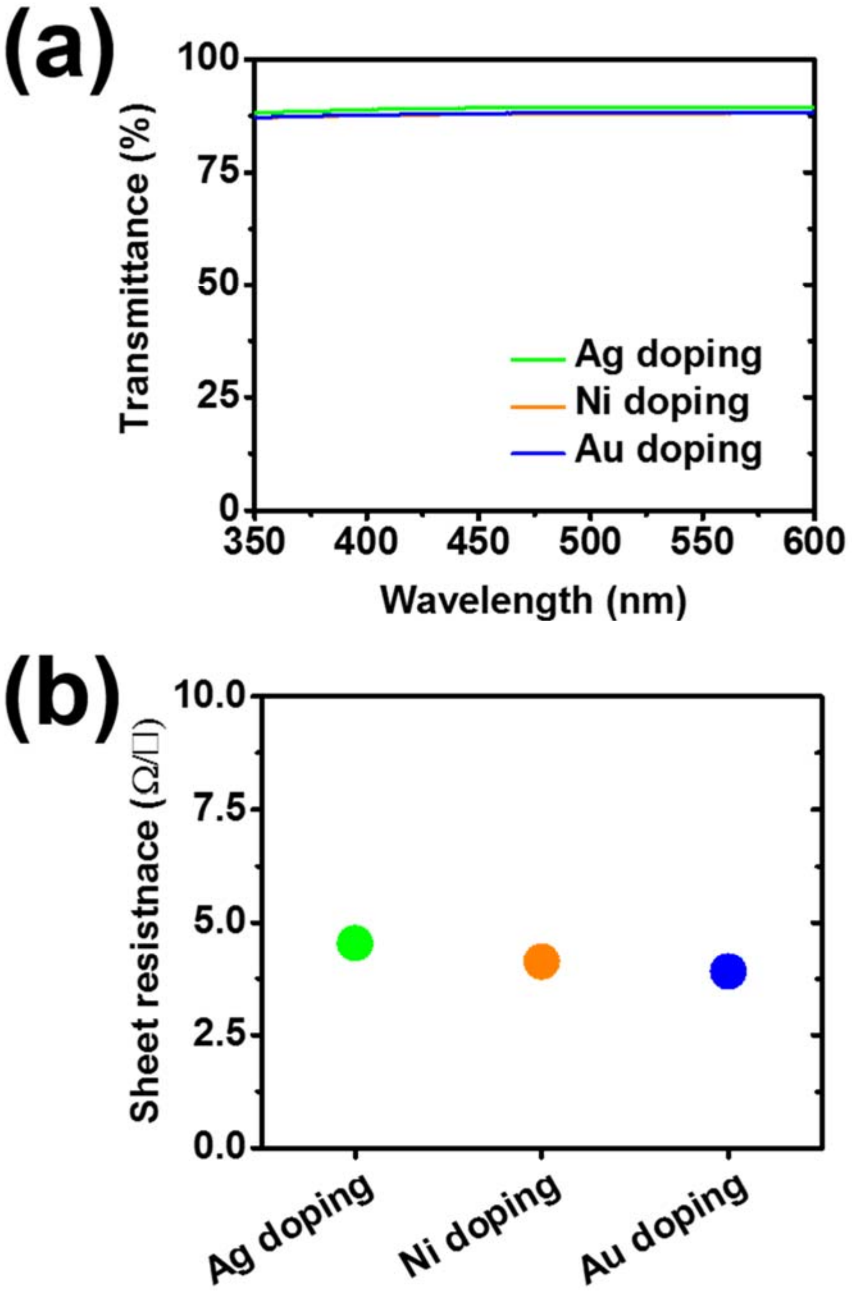

Figure S6. Optical and electrical properties of metal mesh/metal-doped graphene hybrid films: (a) transmittance values and (b) sheet resistance values.
